# Genetic mouse embryo assay: improving performance and quality testing for assisted reproductive technology (ART) with a functional bioassay

**DOI:** 10.1186/s12958-016-0149-x

**Published:** 2016-03-24

**Authors:** Rebecca S. Gilbert, Brandy Nunez, Kumi Sakurai, Thomas Fielder, Hsiao-Tzu Ni

**Affiliations:** Department of Research and Development, Irvine Scientific, 1830 E. Warner Ave, Santa Ana, CA 92705 USA; Transgenic Mouse Facility, University of California Irvine (UCI), 121 Bison Modular, Irvine, CA 92697 USA

**Keywords:** MEA, QC testing, Early developmental markers, Biocompatibility assay, Embryo culture medium

## Abstract

**Background:**

Growing concerns about safety of ART on human gametes, embryos, clinical outcomes and long-term health of offspring require improved methods of risk assessment to provide functionally relevant assays for quality control testing and pre-clinical studies prior to clinical implementation. The one-cell mouse embryo assay (MEA) is the most widely used for development and quality testing of human ART products; however, concerns exist due to the insensitivity/variability of this bioassay which lacks standardization and involves subjective analysis by morphology alone rather than functional analysis of the developing embryos. We hypothesized that improvements to MEA by the use of functional molecular biomarkers could enhance sensitivity and improve detection of suboptimal materials/conditions.

**Results:**

Fresh one-cell transgenic mouse embryos with green fluorescent protein (GFP) expression driven by *Pou6f1* or *Cdx2* control elements were harvested and cultured to blastocysts in varied test and control conditions to compare assessment by standard morphology alone versus the added dynamic expression of GFP for screening and selection of critical raw materials and detection of suboptimal culture conditions. Transgenic mouse embryos expressing functionally relevant biomarkers of normal early embryo development can be used to monitor the developmental impact of culture conditions.

**Conclusions:**

This novel approach provides a superior MEA that is more meaningful and sensitive for detection of embryotoxicity than morphological assessment alone.

## Background

Early detection of anomalies in developing embryos is crucial to successful outcomes in assisted reproduction technology (ART). The one-cell mouse embryo assay (MEA) is routinely performed as the quality control bioassay to assess the conditions to which human embryos will be subjected [1–4].

The in vitro development of one-cell mouse zygotes to the blastocyst stage by 96 h is closely monitored for normal morphology and sufficient formation of expanded/hatching blastocysts (at least 80 % to pass the test) to evaluate the biocompatibility and quality of culture conditions and materials. However, concerns exist due to the insensitivity of MEA to toxic materials and suboptimal culture conditions [5, 6]. Additionally, this bioassay lacks standardization and involves subjective analysis of morphology alone. The 80 % blastocyst rate passing specification for MEA is set according to accepted trending in the industry and is not based on actual statistical criteria.

Growing concerns about detrimental effects of ART on human gametes, embryos, clinical outcomes and long-term health of offspring require improved methods of risk assessment to provide scientifically robust and functionally relevant assays for pre-clinical studies prior to clinical implementation [7–10]. Several attempts have been made to enhance the sensitivity of the MEA, including culturing embryos in smaller groups, reducing the volume of culture medium, removing protein components from the medium, and counting total nuclei per embryo at 96 h for a quantitative comparison of blastocyst grade [11–13]. Given that the quality of embryos varies from harvest to harvest, it is desirable to make improvements to the MEA by use of functional molecular biomarkers to enhance sensitivity to suboptimal materials and conditions and, in turn, increase the safety and successful outcome of in vitro fertilization.

Evidence of dynamic expression and functional profiles of transcription factors during embryogenesis is accumulating. Early developmental genes are known to be differentially expressed starting at the two-cell stage, around the time when gene expression profiles switch from the maternal pattern to the zygotic pattern (zygotic gene activation, or ZGA), continuing through the blastocyst stage and exhibiting localized expression patterns in the developing embryo. *Pou5f1* (previously known as *Oct4*) is a well-described transcription factor known for its role as a pluripotency regulator [14–17]. *Pou5f* is highly expressed starting at the 4- to 8-cell stage after initiation of ZGA and throughout embryonic development. Caudal type homeobox 2 (*Cdx2*) is another early developmental gene that signals the initial stages of cell lineage differentiation in pre-implantation mouse embryos. *Cdx2* expression starts around E3.5 when asymmetric cell divisions in 8- & 16-cell embryos occur, and is tightly restricted to the trophectoderm (TE) [18, 19]. POU5F1 and CDX2 are initially co-expressed in pre-implantation mouse embryos and form a complex for the reciprocal repression of their target genes in embryonic stem cells [20]. During progression from morula to blastocyst, coordinated repression of *Pou5f1* by CDX2 takes place. A regulatory complex formed with CDX2 suppresses expression of *Pou5f1* in the TE, while the expression of *Pou5f1* is maintained within the ICM in the absence of CDX2, thereby establishing localized expression patterns of POU5F1 and CDX2 [21].

The advantages of using mouse embryos for quality control of ART media and materials cannot be ignored. Transgenic mouse technology may provide further advantages by allowing us to generate embryos expressing fluorescent reporter proteins under the control of regulatory elements of early developmental markers, such as POU5F1 and CDX2, for a more sensitive and biologically relevant detection of embryotoxicity.

In this study, we used transgenic mouse embryos expressing green fluorescent protein under control of *Pou5f1* (POU5F1-GFP) or *Cdx2* (CDX2-GFP) regulatory elements and monitored developing embryos with GFP expression patterns in addition to the standard morphology assessment criteria, as a more sensitive means to distinguish suboptimal from optimal embryo culture conditions. We refer to this combination of expression patterns and morphological assessment as the Genetic Mouse Embryo Assay (MEGA™). We found that a high level of fluorescence intensity observed at 48 h (early fluorescence intensity, or EFI) is predictive for the successful development of blastocysts at 96 h. We also show that MEGA outperforms MEA by combining the state of zygotic gene expression and cell lineage differentiation with morphological assessment, and can be applied as a stand-alone in vitro model system of embryonic development to determine biocompatibility of ART media and materials.

## Methods

### Preparation and collection of mouse embryos

Mice were purchased from the Jackson Laboratory (Jax, Bar Harbor, ME) and maintained by the UC-Irvine Transgenic Mouse Facility (TMF). Mouse strain B6C3F1/J was used in studies for standard MEA (no transgene). Mouse strain B6; CBA-Tg (Pou5f1-EGFP) 2Mnn/J carries a randomly-integrated transgene that expresses enhanced GFP (EGFP) under control of the *Pou5f* promoter and distal enhancer. Mouse strain STOCK-Cdx2^tm1Yxz^/J has the GFP coding sequence inserted in-frame, downstream of the third exon of *Cdx2*, by homologous recombination. Mice of either strain were mated with B6D2F1/J mice. The TMF collected zygotes from females the morning after mating were set up, examined each zygote for evidence of fertilization, and supplied all fertilized one-cell embryos in FHM (Millipore, USA, catalog # MR-024-D) to Irvine Scientific. All procedures were conducted with the approval of UCI’s Institutional Animal Care & Use Committee, in accordance with all relevant institutional and federal regulations.

Prior to mating, embryo donor female mice were superovulated by IP injection with 5 IUs of pregnant mare serum gonadotropin (PMSG, NHPP, Torrance, CA) between noon and 2 PM, followed 46–48 h later by an IP injection of 5 IUs of recombinant human chorionic gonadotropin (hCG, NHPP, Torrance, CA), to stimulate the release of oocytes and synchronize estrus cycles. Matings (1 female per male) were set up immediately following the hCG injection. Mating performance of the studs was assessed by recording the presence or absence of a mating plug in each female the next day, just prior to harvesting. Stud males that failed to plug 3 consecutive times were replaced, and all studs were replaced at 10–12 months of age.

Embryo donors were euthanized by CO_2_ inhalation. Oviducts were removed and placed in FHM containing 0.3 mg/ml hyaluronidase (Sigma, St. Louis, MO, catalog # H3884). Cumulus-oocyte complexes were released from the oviducts by tearing open the ampulla and exposed to hyaluronidase just long enough to separate cumulus cells from the oocytes (<5 min). The oocytes were then passed through several washes of FHM to remove hyaluronidase, and observed on an inverted microscope with DIC optics. Oocytes were considered to be fertilized if they had normal morphology and had exactly 2 pronuclei.

### Embryo culture

Fertilized, freshly harvested one-cell mouse embryos (with or without transgene) were cultured in Continuous Single Culture Medium (CSCM) with 5 mg/mL human serum albumin (HSA) with oil overlay (Irvine Scientific). Embryo culture was conducted using either micro-drops (3–4 embryos/20 μL drop) overlayed with 7 mL mineral oil on 60 mm petri dishes (Falcon, #35-1007) or micro-wells (one embryo/10 μL/well or 10 embryos/10 μL/well) overlayed with 9 mL mineral oil on 72-micro-well plates (VWR #82050-710) and incubated at 37 °C, 5 % CO_2_ in air (21 % O_2_) for 96 h. Culture medium in dishes/plates was pre-equilibrated overnight in the 37 °C incubator with 5.0 – 5.5 % CO_2_ in air (21 % O_2_) prior to addition of freshly harvested and washed one-cell embryos. Freshly harvested one-cell mouse embryos were pooled, washed three times in pre-equilibrated culture medium and randomly distributed into the respective test conditions, then returned to the incubated at 37 °C, 5 % CO_2_ in air (21 % O_2_) for up to 96 h with no medium change (uninterrupted). Development rates were evaluated at 48 h and/or 96 h.

### Standard mouse embryo assay

The sensitivity of different culture set up methods was initially evaluated using CSCM-C that was adulterated with addition of formaldehyde (Sigma), which is typically used for proficiency testing, at 0 to 7× concentration (1 × = 9.3 × 10^−5^% formaldehyde) and set up in parallel using micro-drop or micro-well assays (as described above). Fresh one-cell mouse embryos (B6D2F1 × B6C3F1, *n* = 33 per condition) were used for this assay. Blastocyst development rates (expanded and hatching blastocysts) were observed and recorded at 96 h.

### Genetic mouse embryo assay (MEGA™)

Sensitivity of MEGA to suboptimal conditions was determined through experiments that were conducted using a dose response of previously identified detrimental (PID) oil vs. control oil conditions (Irvine Scientific, 2 lots oil, *n* = 30 to 90 embryos per condition, 3 experiments). A case study was then conducted in one experiment, which consisted of screening 5 lots of human serum albumin (HSA) in parallel (including control and test lots) to evaluate variability between lots (*n* = 43 embryos per treatment condition using micro-drop method).

Embryos were graded for morphology at 48 h (as < 8-cell or ≥ 8-cell stage) and fluorescence intensity (termed early fluorescence intensity or EFI) of POU5F1-GFP expression was observed with fluorescent microscope (Olympus IX51, 480nmEx/535/550nmEm) and scored on a scale of 0–3 at 48 h. The percentages of embryos with a low EFI of 0 to 1 (EFI 0–1) or a high EFI of 2 to 3 (EFI 2–3) were calculated and these values were compared to the blastocyst development rates of the respective conditions to determine correlation between high EFI with % blastocyst at 96 h. Embryos were also graded at 96 h as ≤ blastocysts (up to blastocyst with small blastocoel cavity), or expanded (>50 % blastocoel cavity, large and well-expanded) and hatching blastocysts (with a herniation in the zona pellucida (ZP) or hatched) and calculated as a percent of total embryos analyzed. Similar experiments were conducted using the mice transgenic for *Cdx2*-*GFP*. The ICM-specific expression of POU5F1-GFP and TE-specific expression of CDX2-GFP at the advanced blastocyst stages were examined at 96 h for further verification of normal blastocyst development. Two-way ANOVA followed by Dunnett’s multiple comparisons test was performed using GraphPad Prism version 6.05 (GraphPad Software, www.graphpad.com).

## Results

**Fig. 1:**
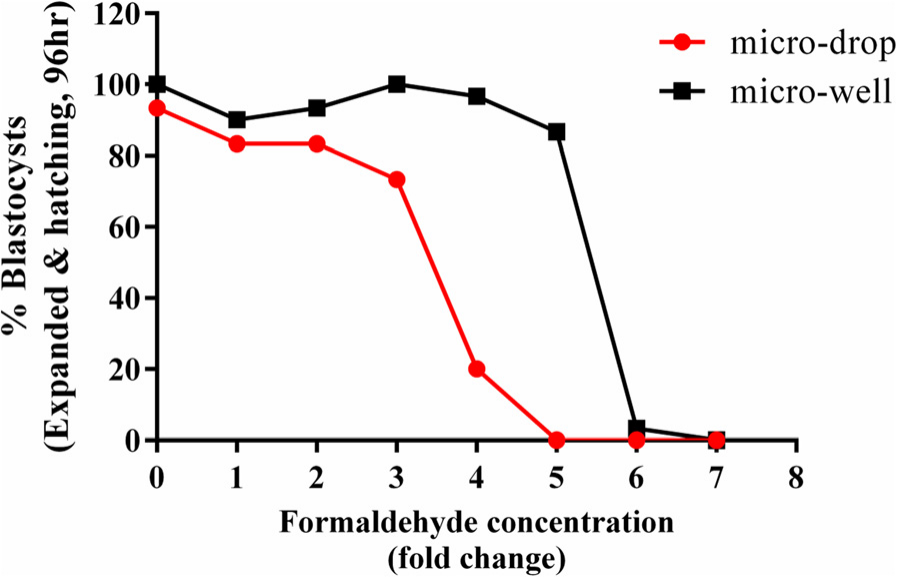
Detection of toxicity by two mouse embryo culture methods. To determine sensitivity of two culture methods—micro-drop (red) or micro-well (black), adulterated media containing formaldehyde (from 1 to 7× concentrated, where 1 × = 9.3 × 10^−5^%) were used in parallel to culture fresh one-cell mouse embryos (B6D2F1 × B6C3F1). Development of mouse embryos was monitored by morphology at 96 h after uninterrupted culture for % blastocyst rates (including expanded and hatching/hatched, *n* = 33)

To determine the impact of culture methodology on assay sensitivity, we examined two embryo culture methods—micro-well and micro-drop methods—for relative sensitivity in detecting toxicity of materials and suboptimal culture conditions. Adulterated media containing formaldehyde (from 1 to 7× concentrated, where 1 × = 9.3 × 10^−5^ %) was used to culture fresh one-cell mouse embryos (B6D2F1 × B6C3F1) with micro-drop or micro-well in parallel (Fig. 1). A passing development rate of at least 80 % at 96 h was observed with 2 fold concentrated (2×) formaldehyde with micro-drops (3–4 embryos/20 μL drop) while embryos cultured in micro-wells (10 embryos/10 μL/well) tolerated up to 5× formaldehyde, demonstrating that use of the micro-drop method is more sensitive to toxicity than the micro-well method. The micro-drop method was therefore used throughout the rest of this study, unless otherwise noted.

**Fig. 2:**
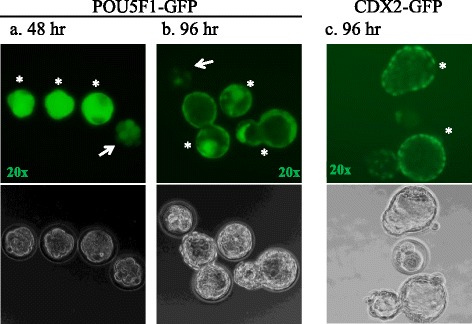
POU5F1-GFP and CDX2-GFP expression during early embryogenesis in vitro. Development of 2PN zygotes in control medium was monitored for morphology and expression up to 96 h. POU5F1-GFP expression at 48 h (*a*) was used to score embryos on a scale of 0 to 3 for early fluorescence intensity (EFI): low EFI (score 0–1, arrow) or high EFI (score 2–3,*). Three embryos exhibited high EFI of POU5F1-GFP at 48 h (*a*) and subsequently developed into expanded and hatching blastocysts (*b*) with expression localized to the ICM (*), compared to one embryo (arrow) with low EFI which failed to develop to the blastocyst stage. Similarly, CDX2-GFP embryos that developed to the expanded/hatching blastocyst stages at 96 h express the normal localization pattern of CDX2 in the TE (c*)

In order to further improve the sensitivity of the MEA, we integrated mouse embryos with a reporter protein linked to an endogenously expressed early developmental regulator. We hypothesized that *Pou5f1*-*GFP* zygotes with 2 pronuclei (2PN) produced by mating transgenic mice strain B6; CBA-Tg (Pou5f1-EGFP) 2Mnn/J with B6D2F1/J can be cultured in vitro and monitored at 48 h and 96 h to assess their development (Fig. 2). Consistent with previous reports, we observed expression of POU5F1-GFP in blastomeres of cleavage stage embryos as early as 48 h, which was subsequently down regulated and localized to the ICM [14, 15] of expanded blastocysts (Fig. 2a). Some *Pou5f1*-*GFP* embryos failed to fully develop into blastocysts or did not reach the expanded blastocyst stage at 96 h. Notably, POU5F1-GFP was expressed to varying degrees among embryos at 48 h, and those embryos showing a high EFI were shown to correlate with successive development to the advanced blastocyst stages (expanded/hatching), while embryos with a low EFI (Fig. 1a & b, arrows) exhibited arrested or delayed development.

One-cell (2PN) transgenic CDX2-GFP zygotes were also obtained, cultured in control medium and observed for expression and development up to 96 h (Fig. 2c). CDX2-GFP expression was localized to the TE in expanded blastocysts at the 96 h time point, confirming normal blastocyst development [21]. However, overall weak fluorescent intensity at 48 h (~8-cell cleavage) made scoring difficult, and the CDX2-GFP embryos did not exhibit a strong correlation between EFI and ongoing blastocyst development, therefore subsequent studies were limited to POU5F1-GFP embryos.

**Fig. 3:**
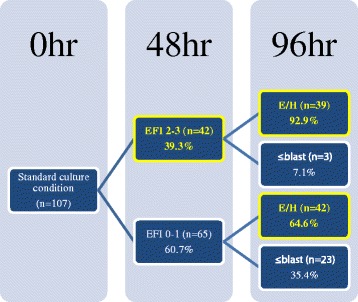
Micro-well culturing POU5F1-GFP expressing embryos. 2PN zygotes (*n* = 107) expressing *Pou5f1*-*GFP* were cultured individually in control medium with micro-well method (one embryo/10 μL/well) and monitored for morphology and GFP expression up to 96 h. Developmental progress of individual embryos was shown as a hierarchical diagram. *Pou5f1*-*GFP* embryos were evaluated at 48 h and grouped as EFI 0–1 (score 0–1) or EFI 2–3 (score 2–3). Their successive development was monitored and graded at 96 h with detection of POU5F1-GFP and divided into two groups: expanded and hatching/hatched blastocysts (E/H, yellow) or ≤ blastocysts (white)

Next we monitored and tracked *Pou5f1*-*GFP* embryo development and POU5F1-GFP expression up to 96 h by culturing the embryos in control medium in micro-well plates (one embryo/10 μl/well) to determine if there was a correlation between early *Pou5f1*-*GFP* expression and embryonic development (Fig. 3). POU5F1-GFP expression at 48 h (as shown Fig. 2a) was used to score embryos on a scale of 0 to 3 as early fluorescence intensity (EFI) and grouped into low EFI (EFI 0–1) or high EFI (EFI 2–3) (Fig. 3, 48 h). Successive development of *Pou5f1*-*GFP* embryos were monitored and graded at 96 h with localization pattern of POU5F1-GFP and grouped according to their morphological stage: expanded and hatching/hatched blastocysts (E/H, yellow) or ≤ blastocysts (white). Notably, 92.9 % of the embryos that exhibited a high EFI at 48 h (39.3 % of total embryos tested, *n* = 107) developed into E/H blastocysts by 96 h while 64.6 % of the embryos with low EFI at 48 h (60.7 % of total embryos) developed into E/H blastocysts by 96 h. Overall, the total % Blastocyst (E/H) development by morphology alone was 75.7 % (*n* = 81) at 96 h.

**Fig. 4:**
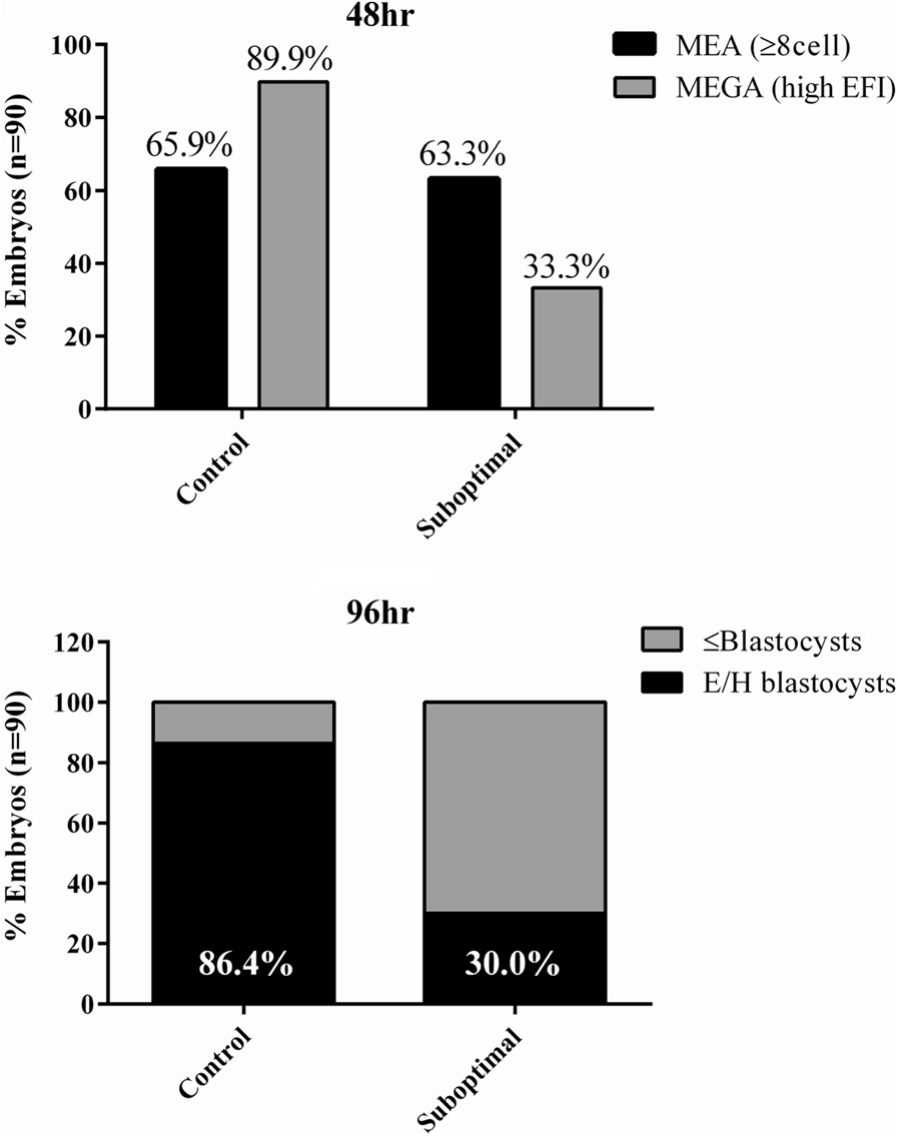
Double-blinded assessment of *Pou5f1*-*GFP* embryo development. Control and suboptimal (10 % PID oil) culture conditions were tested with micro-drop method for effect on *Pou5f1*-*GFP* embryo development and gene expression. *Pou5f1*-*GFP* embryos were evaluated at 48 h for morphology (≥8 cell) and expression (MEGA, high EFI 2–3), and subsequently assessed at 96 h for blastocyst development with localized expression of POU5F1-GFP, shown as % Embryos

*Pou5f1*-*GFP* embryos cultured in micro-drops overlayed with control and suboptimal (10 % PID) mineral oil were used in a double-blinded assessment of MEA (morphology alone) and MEGA (EFI of POU5F1-GFP expression) to determine the level of sensitivity for detecting suboptimal conditions (Fig. 4). Double-blinded assessment of embryos was conducted at 48 h by morphology (reaching at least 8-cell stage) and by EFI (high EFI). At 48 h morphological development to ≥8 cells stage of embryos in control and suboptimal conditions were comparable at 65.9 % and 63.3 %, respectively, whereas 89.8 % of control embryos expressed high EFI compared to only 33.3 % for embryos under suboptimal conditions, suggesting that POU5F1-GFP expression is a more sensitive indicator of normal embryo development. Subsequent blastocyst development rates at 96 h (graded by morphology and localized expression of POU5F1-GFP) were 86.4 % for control and 30.0 % for suboptimal condition, results similar to those for high EFI rates at 48 h, further demonstrating the predictive value of EFI at 48 h and sensitivity of POU5F1-GFP expression over morphology alone.

**Fig. 5:**
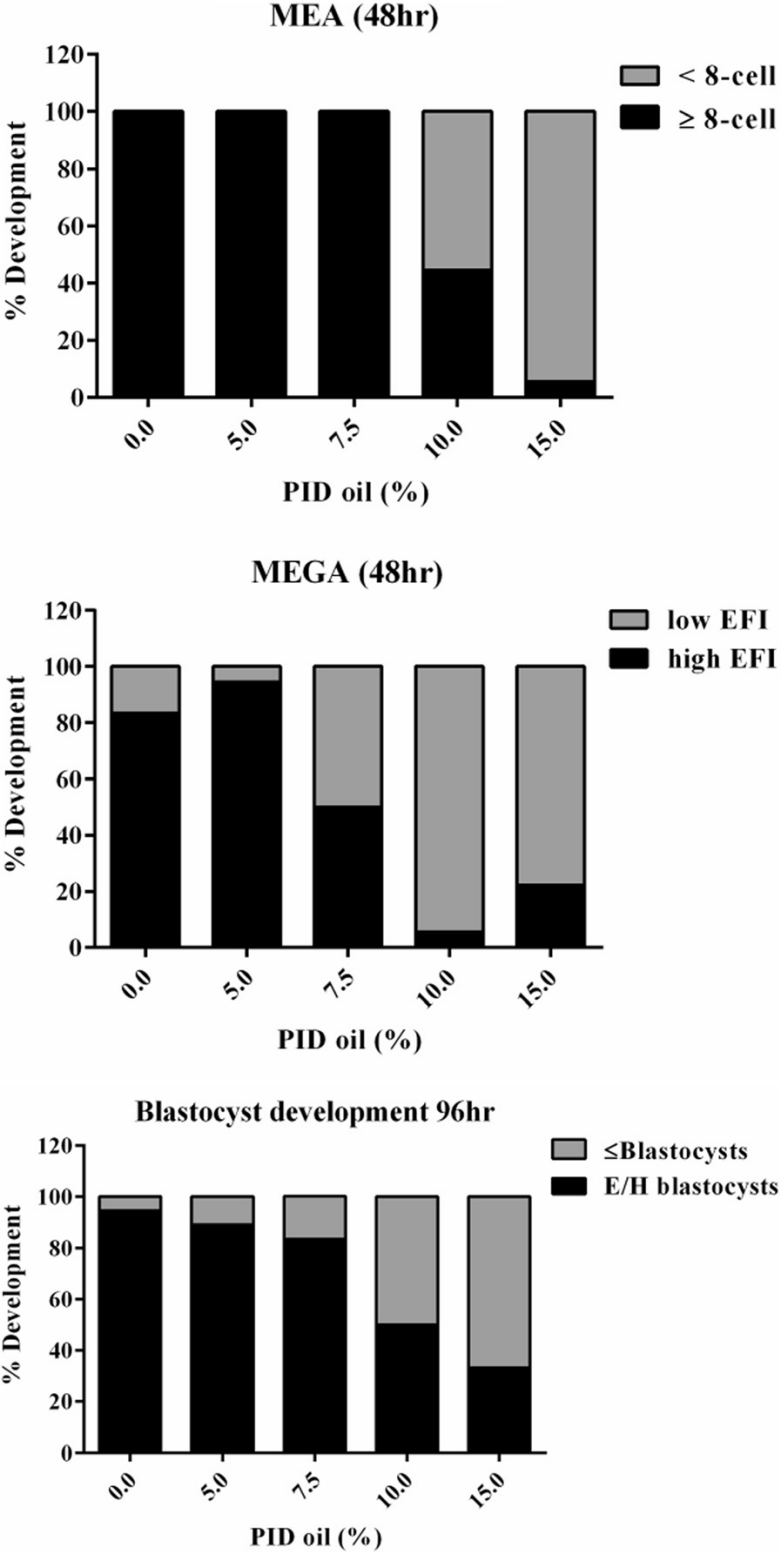
Oil dose–response. *Pou5f1*-*GFP* embryos (*n* = 20 per condition) were cultured under PID oil (0, 5, 7.5, 10, or 15 %) and evaluated for embryo development/expression at 48 h (8-cell or EFI) and 96 h (E/H blastocysts)

To assess the responsiveness of MEGA as a stand-alone bioassay, *Pou5f1*-*GFP* embryos cultured by micro-drop assay were evaluated in a dose–response to PID oil (0–15 %) and evaluated for development (morphology) and expression at 48 h and 96 h (Fig. 5). At 48 h toxicity was shown at 10 % PID oil by morphology, but EFI was more sensitive in detecting toxicity at 7.5 %, supporting previous findings. The blastocyst development rates at 96 h (which included localization of expression) showed toxicity starting at the 10 % PID concentration.

**Fig. 6:**
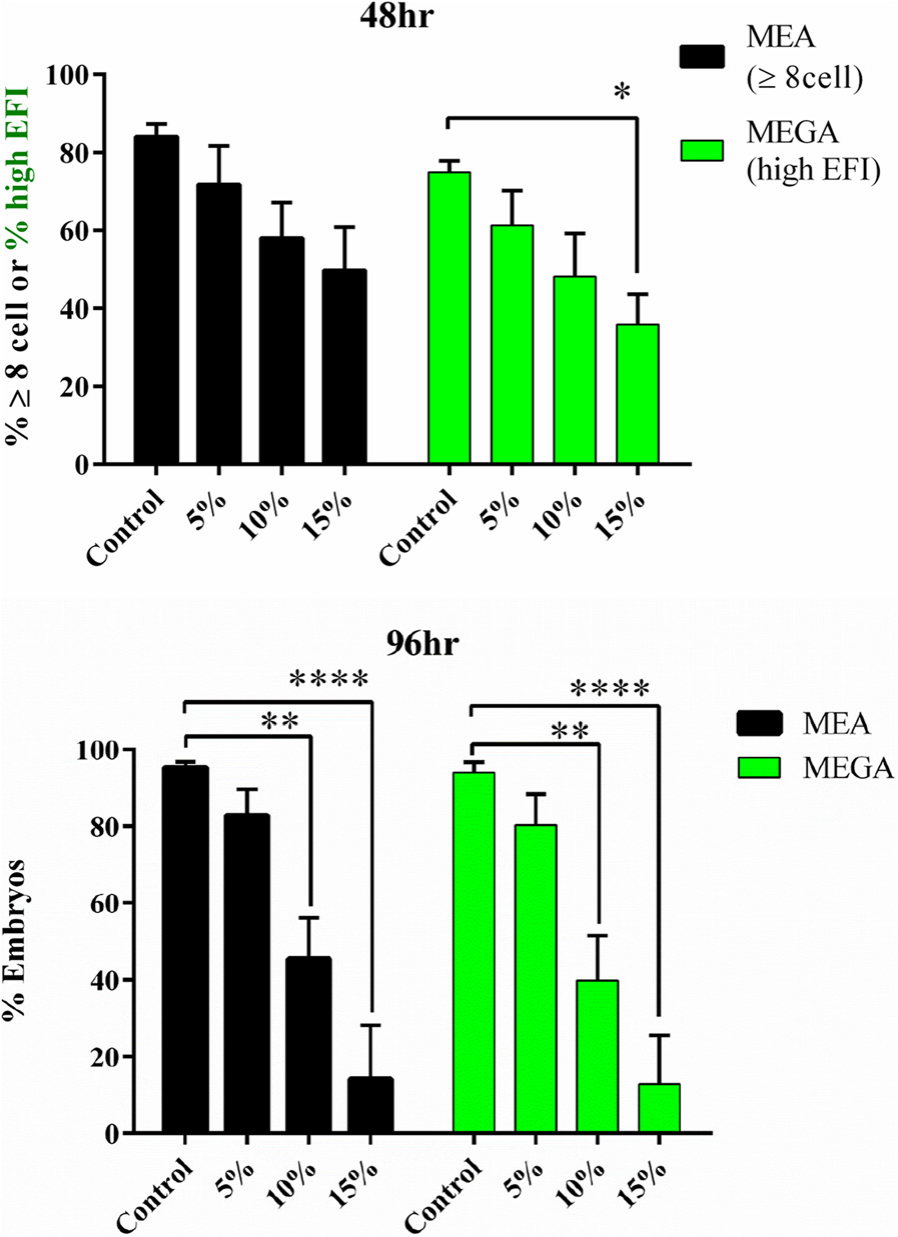
Oil dose–response and sensitivity of MEGA for detection of suboptimal conditions. The sensitivity of *Pou5f1*-*GFP* embryos to culture conditions was further assessed with various doses of PID oil. *Pou5f1*-*GFP* embryos cultured under oil overlays containing 0 (control), 5, 10, or 15 % PID oil were evaluated for embryo development/expression at 48 h (8-cell or EFI) and 96 h (E/H) (3 experiments). Statistical analysis: 2-way ANOVA with multiple comparisons. *p < 0.05, **p < 0.005, ****p < 0.0001. Error bars: mean ± SEM

Additional experiments were conducted with *Pou5f1*-*GFP* embryos under suboptimal conditions of a dose–response with PID oil. *Pou5f1*-*GFP* embryos from three different harvests were cultured in vitro under adulterated mineral oil (5 %, 10 %, or 15 % PID oil) and graded by MEA and MEGA. *Pou5f1*-*GFP* embryos showed dose–response of development and POU5F1-GFP expression and were reproducible with the concentrations tested (Fig. 6). At 48 h, the % high EFI was significantly lower for MEGA embryos in 15 % PID oil condition (*p* < 0.05) and at 96 h the % blastocyst rates for both MEA and MEGA were significantly lower for 10 % PID oil (*p* < 0.005) and 15 % PID oil (*p* < 0.0001).

To further demonstrate the sensitivity of MEGA to embryo culture conditions, a case study of various lots of HSA raw material was conducted using *Pou5f1*-*GFP* embryos (*n* = 43 per condition) evaluating morphology and POU5F1-GFP expression at 48 and 96 h. The results in Table 1 show no difference by 48 h morphology (at least 95 % of embryos reached at least 8 cell stage), but high EFI suggests 2 lots of HSA (lots A and D) may be suboptimal. The subsequent blastocyst rates (E/H = expanded and hatching) at 96 h were lowest for the two corresponding lots of HSA reaching 72.7 % (failing) and 83.7 % for HSA-A and HSA-D, respectively, compared to the other lots which resulted in >85 % expanded/hatching blastocysts, demonstrating the predictive value and sensitivity of EFI over morphology alone. Taken together, these data demonstrate that MEGA may have greater sensitivity than standard MEA in addition to an early predictive value of suboptimal conditions at 48 h which are confirmed by subsequent rates of normal embryo development at 96 h.

**Table 1:** HSA Case study

Culture conditions	48 h		96 h
	≥8-cell morphology	High EFI	^a^E/H Blastocysts
HSA-A	95.5	86.4	72.7
HSA-B	95.3	90.7	86.0
HSA-C	97.7	93.0	93.0
HSA-D	97.7	81.4	83.7
HSA-E	95.3	95.3	95.3

^a^E/H, expanded and hatching blastocysts

## Discussion

The culture method used for the mouse embryo assay can impact the sensitivity of detecting toxic substances, as demonstrated by use of the micro-drop assay. Possible explanations for the enhanced sensitivity of the micro-drop assay include: 1) availability of less autocrine/paracrine factors in the microenvironment as a result of lower embryo/volume ratio and separation of embryos resting on the flat surface under micro-drop (compared to the micro-well assay with conical shape, closer proximity of embryos, higher embryo/culture volume ratio and availability of more autocrine/paracrine factors which may benefit embryo development and mask environmental toxicity), 2) the relative surface area exposure of oil (and potential toxins therein) to embryo culture medium is greater in micro-drop method.

By using the more sensitive micro-drop culture method and employing transgenic mouse embryos expressing POU5F1-GFP, we demonstrated MEGA to be an improved bioassay with increased sensitivity to toxic materials and suboptimal culture conditions, with early predictive values of development, using early fluorescence intensity (EFI) at 48 h, in addition to standard morphological evaluation of blastocysts at 96 h. Furthermore, unlike the standard MEA, a fluorescence-based bioassay can be assessed quantitatively and standardized for consistency. Our findings suggest MEGA can be applied as a stand-alone in vitro quality-control system. The ability to observe the state of zygotic gene expression and obtain more detailed morphological assessments (e.g., by monitoring normal/expected localization of expression) adds versatility to the bioassay. Timely expression of selected early development regulator *Pou5f1* in transgenic mouse embryos was correlated with embryonic development to blastocysts and provided sensitive screening for suboptimal culture conditions and materials.

It should be noted that while CDX2-GFP was expressed properly in TE of blastocysts, where TE development starts around E3.5 (or 60 h after fertilization), the corresponding EFI at 48 h could not be accurately scored because CDX2-GFP expression started at a time point later than 48 h. To address this issue, we plan to evaluate other fluorescent reporters and perform time-lapse fluorescence microscopy on individual embryos. Larger studies will need to be conducted with key markers to build sufficient data for statistical analysis to define the quantitative acceptance criteria for practical application.

Additional enhancements to the MEGA protocol might be achieved by combining two or more different colors of fluorescent reporters in a single mouse strain, to allow simultaneous monitoring of more than one early development regulator or cell lineage. Furthermore, we hypothesize that sensitivity of MEGA can be enhanced by integrating other genes which are active during pre-implantation and potentially impacted by culture conditions or ART materials.

## Conclusions

These studies demonstrate a proof of principle that transgenic mouse embryos expressing POU5F1-GFP, a functionally relevant biomarkers of normal early development, can be used in combination with morphological assessment as an enhanced assay (MEGA®) to detect embryotoxicity and identify the developmental impact of materials and culture conditions. Our data suggest that the addition of functional molecular biomarkers to morphology can improve quality control assessment of biocompatibility of ART media and materials prior to clinical implementation and thereby better address potential impact to ongoing embryo development, ART outcomes and risks to the long-term health of offspring. Further studies are required to define comprehensive molecular indicators of early development that may benefit human reproductive studies including ART, gamete development, and stem cell research.

## Abbreviations

ART: assisted reproductive technology
E/H: expanded and hatching
EFI: early fluorescence intensity
GFP: green fluorescent protein
HSA: human serum albumin
MEA: mouse embryo assay
MEGA: genetic mouse embryo assay
PID: previously identified detrimental
TE: trophectoderm
ZGA: zygotic gene activation
